# In silico genome-wide miRNA-QTL-SNPs analyses identify a functional SNP associated with mastitis in Holsteins

**DOI:** 10.1186/s12863-019-0749-5

**Published:** 2019-05-16

**Authors:** Qiang Jiang, Han Zhao, Rongling Li, Yaran Zhang, Yong Liu, Jinpeng Wang, Xiuge Wang, Zhihua Ju, Wenhao Liu, Minghai Hou, Jinming Huang

**Affiliations:** 1 Dairy Cattle Research Center, Shandong Academy of Agricultural Science, Jinan, 250131 Shandong People’s Republic of China; 2 College of Life Sciences, Shandong Normal University, Jinan, 250014 Shandong China

**Keywords:** Dairy cattle, SNP, Bta-miR-2899, SPI1, Mastitis

## Abstract

**Background:**

Single-nucleotide polymorphisms (SNPs) in microRNAs (miRNAs) and their target binding sites affect miRNA function and are involved in biological processes and diseases, including bovine mastitis, a frequent inflammatory disease. Our previous study has shown that bta-miR-2899 is significantly upregulated in the mammary gland tissue of mastitis-infected cow than that of healthy cows.

**Results:**

In the present study, we used a customized miRNAQTLsnp software and identified 5252 SNPs in 691 bovine pre-miRNAs, which are also located within the quantitative trait loci (QTLs) that are associated with mastitis and udder conformation-related traits. Using luciferase assay in the bovine mammary epithelial cells, we confirmed a candidate SNP (rs109462250, g. 42,198,087 G > A) in the seed region of bta-miR-2899 located in the somatic cell score (SCS)-related QTL (Chr.18: 33.9–43.9 Mbp), which affected the interaction of bta-miR-2899 and its putative target *Spi-1 proto-oncogene* (*SPI1*), a pivotal regulator in the innate and adaptive immune systems. Quantitative real-time polymerase chain reaction results showed that the relative expression of *SPI1* in the mammary gland of AA genotype cows was significantly higher than that of GG genotype cows. The SNP genotypes were associated with SCS in Holstein cows.

**Conclusions:**

Altogether, miRNA-related SNPs, which influence the susceptibility to mastitis, are one of the plausible mechanisms underlying mastitis via modulating the interaction of miRNAs and immune-related genes. These miRNA-QTL-SNPs, such as the SNP (rs109462250) of bta-miR-2899 may have implication for the mastitis resistance breeding program in Holstein cattle.

**Electronic supplementary material:**

The online version of this article (10.1186/s12863-019-0749-5) contains supplementary material, which is available to authorized users.

## Background

Mastitis is a common, complex and devastating inflammatory disease, which is caused by pathogenic infections and can result in significant economic losses to the dairy industry [1, 2] Molecular breeding is a technique used to increase the resistance ability to mastitis via expanding the favorable allele frequency in dairy cattle populations and is considered a feasible and long-term strategy. Evidence has shown that the consistent progress in mastitis resistance can be achieved by following genetic estimation and selection of sire, generating favorable udder heath phenotypes, such as increased udder conformation score of cows, decreased incidence of mastitis, and milk somatic cell score (SCS) [3]. In the genetic evaluation, the SCS trait is practical indicator because of its strong positive relationship with the presence of mastitis, and accessbility of measurement superior to mastitis in cow [4]. Further, SCS trait has obtained dramatic response to genomic selection of bull, resulting in rapid genetic improvement in recent years [5]. However, the recent development of genomic selection programs are based on genotypes of single-nucleotide polymorphism (SNP), most of which have small effects and are distributed evenly across all chromosomes, and are non-functioning markers. The accuracy and reliability of genomic prediction are expected to enhance and the number of SNP needed for evaluation is expected to decrease if the functional or causative SNPs are enrolled in the evaluation [6, 7].

MicroRNAs (miRNAs) are class of small, endogenous, non-coding RNAs that are critical and versatile regulators of inflammation-, immune-, and defense-related gene expression levels during infection with mastitis in dairy cattle [8–10]. A number of studies have revealed that miRNAs are the critical players during host innate immune response to various bacterial infections [8–14]. Furthermore, most of the quantitative trait loci (QTLs) for mastitis resistance on chromosomes have been reported for different cattle populations [15]. However, the adequate application of these resources remains a challenge. A total of 2.44 million SNPs (Bos_taurus_UMD_3.1 reference assembly) [16], 1025 miRNAs (http://www.mirbase.org/), and 120,122 QTLs (https://www.animalgenome.org/cgi-bin/QTLdb/index) are deposited in the dbSNP, miRNA and Animal QTL databases, respectively. However, the association of SNPs with miRNA function and mastitis susceptibility have not been elucidated. Therefore, narrowing down the SNPs to the candidate functional variants for further study is an important step. We designed the miRNAQTLsnp software to search functional miRNA SNPs involved in mastitis by integrating available bovine SNPs, miRNAs, and QTL data. Our previous study has revealed that bta-miR-2899 is differentially expressed between the cow’s mastitic mammary gland tissues and healthy tissues [10]. Based on our previous study and bioinformatics of miRNAQTLsnp, we speculated that a candidate SNP (rs109462250, g. 42,198,087 G > A) is mapped on the seed region of bta-miR-2899, and SCS-related QTL (Chr.18: 33.9–43.9 Mbp) will affect the binding of miR-2899 and its putative target *Spi-1 proto-oncogene* (*SPI1*, also named as PU.1). *SPI1* is an E26 transformation-specific family transcription factor that is required for the development of immune system [17] and is a critical regulator for modulation of monocyte/macrophage differentiation [18].

Identification of miRNA target gene and miRNA-related SNPs associated with inflamation and immune responses is essential to elucidate the molecular mechanisms underlying mastitis susceptibility. The present study aimed to achieve the following: (1) to identify the genome-wide miRNA- and mastitis-related SNPs; (2) to confirm whether the candidate SNP in the seed region of bta-miR-2899 will affect the binding of bta-miR-2899 and the bovine *SPI1* gene and its relationship with mastitis susceptibility.

## Results

### Identification of SNPs located within miRNAs and mastitis-related QTLs

**Fig. 1:**
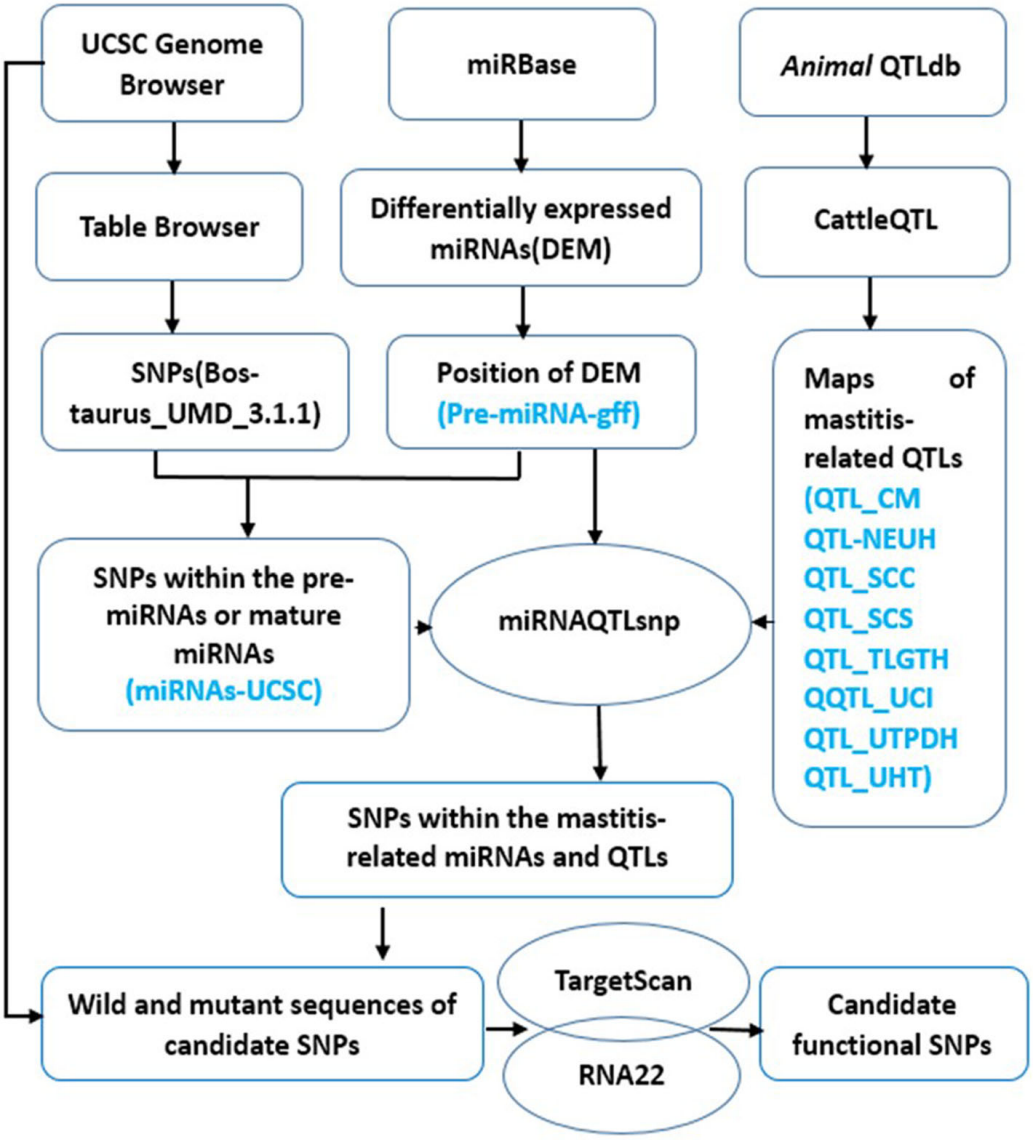
Workflow diagram for identification of miRNA- and mastitis-QTL-related SNPs. The Perl script of miRNAQTLsnp software was shown in the Additional file 1

By using our customized miRNAQTLsnp software (Fig. 1 and Additional file 1), we identified 5252 SNPs (including indel polymorphisms) in 691 bovine pre-miRNAs. Among these SNPs, 1662 were located in 497 mature miRNAs and 617 SNPs in the seed sequences of 331 bovine mature miRNAs (Additional file 2: Table S1). Furthermore, through mapping of SNPs onto miRNAs and within the mastitis-related QTLs, including clinical mastitis (CM), somatic cell count (SCC), SCS, neutrophil count (NEUT), udder composite index (UCI), udder depth (UDPTH), udder height (UHT), and teat length (TLGTH). We detected 2912 SNPs located in the known pre-miRNAs, 8 mastitis-related QTLs, and 325 SNPs harbored in the seed sequences of miRNAs and 8 mastitis-related QTLs (Table 1 and Additional file 3: Table S2).

**Table 1:** SNPs located within the bovine miRNAs and mastitis-related QTLs

	CM	SCC	SCS	NEUT	UCI	UDPTH	UHT	TLGTH
SNPs in pre-miRNAs	270	70	1366	23	203	435	190	355
Number of pre-miRNAs	49	10	169	3	37	49	28	60
SNPs in miRNA seed region	28	9	168	1	28	50	20	21
Number of chromosomes	18	6	23	1	9	12	7	9

*CM* Clinical mastitis, *SCC* Somatic cell count, *SCS* Somatic cell score, *NEUT* Neutrophil count, *UCI* Udder composite index, *UDPTH* Udder depth, *UHT* Udder height, and *TLGTH* Teat length

### Identification of SNP and computational prediction of miRNAs targeting the 3′-UTR of *SPI1* gene

**Fig. 2:**
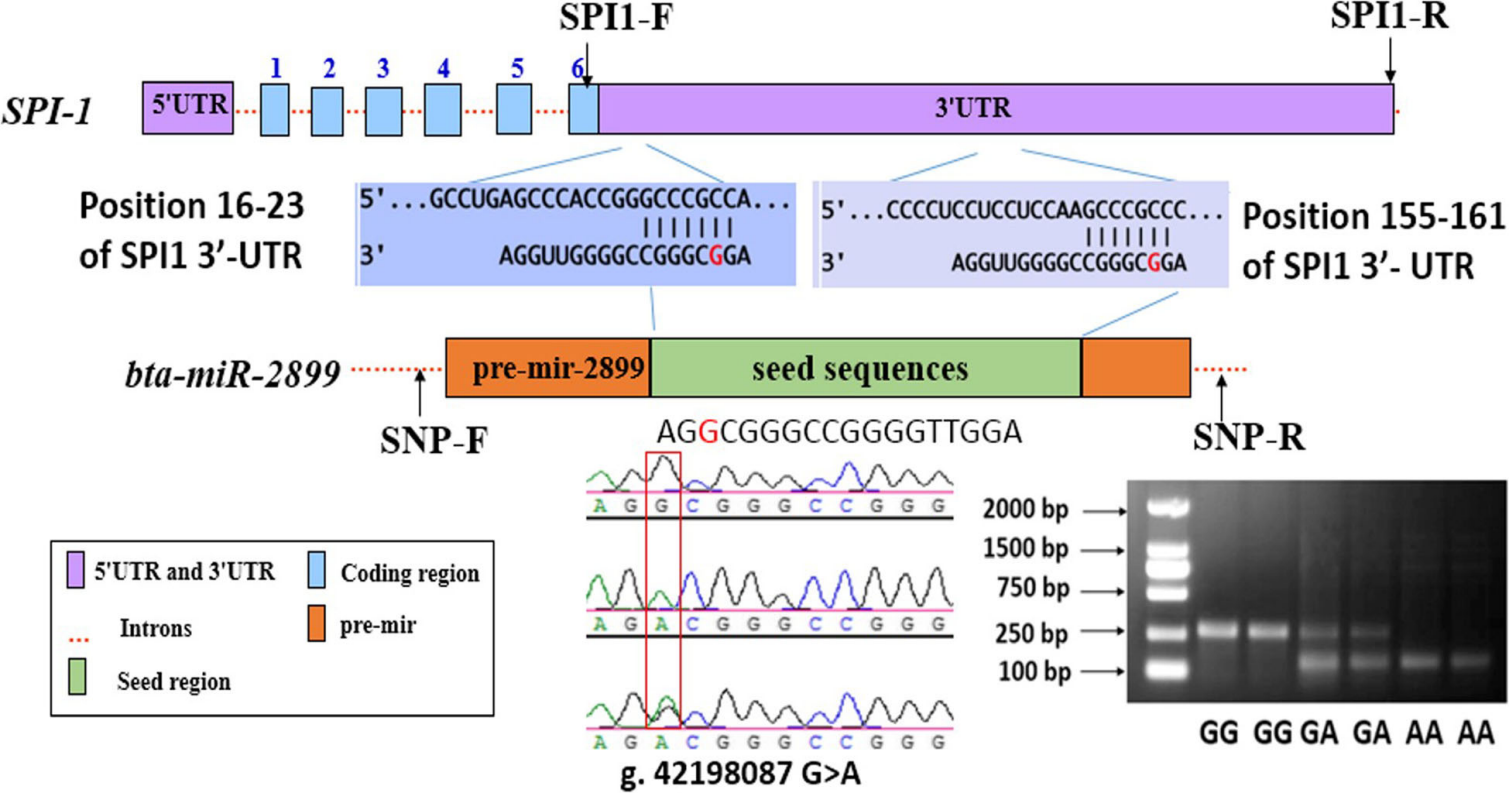
Identification of SNP g. 42,198,087 G > A and two putative binding sites of bta-miR-2899 and SPI1. WT sequence of bta-miR-2899 featured two high-likelihood binding sites to 3′-UTR of the *SPI1* (position 16–23; positions 155–161); the MT sequence of bta-miR-2899 cannot generate any binding site

We observed that the candidate SNP (rs109462250) is located in the seed sequence of bta-miR-2899 and within the SCS-related QTL region (Chr.18: 33.9–43.9 Mbp). This candidate SNP was predicted to alter the binding of 3′-untranslated regions (3′-UTR) of SPI1 with bta-miR-2899 and it was selected as an example for the next analysis. As shown in Fig. 2, direct sequencing identified the SNP (g. 42,198,087 G > A, rs109462250) in the seed region of bta-miR-2899. Mutation generated a restriction endonuclease (FauI) binding site. The PCR products were digested by FauI, which was separated by the agarose gel, and showed three genotypes (GG, 268 bp; AG, 268 + 133 + 135 bp, and AA, 133+ 135 bp).

To identify whether the WT and MT bta-miR-2899 potentially regulates the expression of *SPI1*, we integrated the results of two software programs to detect the potential target gene of bta-miR-2899. As shown in Fig.2, the WT sequence of bta-miR-2899 exhibited two high-likelihood binding sites to 3′-UTR of *SPI1*, whereas the MT sequence of bta-miR-2899 cannot generate any binding site.

### Expression of SPI1 mRNA in the mammary gland tissues of cows with different genotypes

**Fig. 3:**
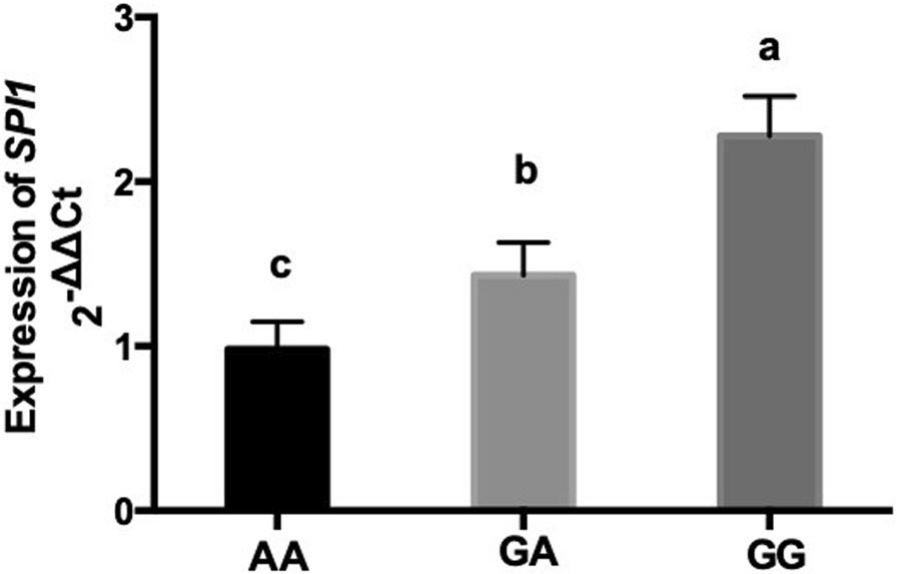
Relative expression of *Spi-1 proto-oncogene* (*SPI1*) mRNA in the mammary gland tissues of cows with different genotypes in the SNP (g. 42,198,087 G > A) locus. Relative expression is represented as mean ± SD; vertical bars denote the SD. Different superscripts (**a**, **b** and **c**) in vertical error bars represent significant difference (*P* < 0.05)

To investigate whether *SPI1* will be expressed in the mastitic mammary glands, using quantitative real-time PCR (Q-PCR), we compared the relative expression of *SPI1* mRNA in the mastitic mammary gland tissues of cows with three genotypes. As a result, the relative expression of *SPI1* mRNA in cows with the AA genotype was significantly higher (*P* < 0.05) than the cows with GG and GA genotypes (Fig. 3).

### Activity analysis of *SPI1* 3′-UTR targeted by bta-miR-2899

**Fig. 4:**
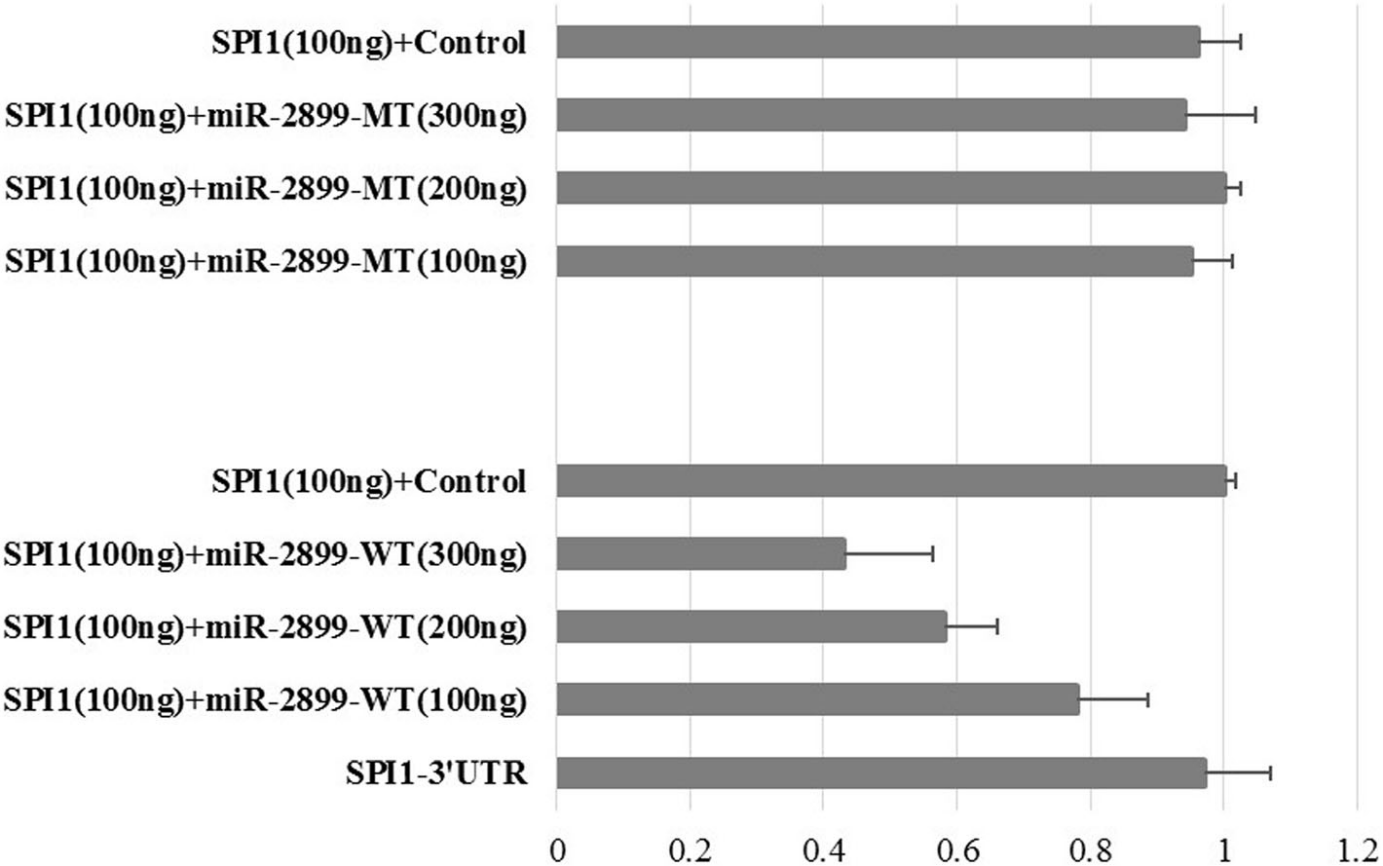
Differential expression ratios of Luc reporters carrying WT or MT miR-2899 with different doses of *Spi-1 proto-oncogene* (*SPI1*) gene. A luciferase reporter vector containing the SPI1 mRNA 3′-UTR co-transfected with ß-gal in MAC-T. WT, g. 42,198,087 G > A-GG; MT, g. 42,198,087 G > A-AA. MiR-2899 (CCS-bta-223-MR04) at quality of 100 ng to 300 ng dose dependently repressed the reporter activities. Mock-transfected cells treated with the pMIR-REPORT vector alone. CmiR001-MR04 served as a scrambled control. Data are representative of four separate experiments (mean ± SD)

To validate whether *SPI1* is directly targeted by bta-miR-2899 in cow, the WT *SPI1* 3′-UTR sequence was cloned to a luciferase reporter gene in a luciferase expression vector and transfected into bovine mammary epithelial cells (MAC-T). Dose-dependent repression of the luciferase activity was observed when concentrations of WT bta-miR-2899 were increased and co-transfected with the luciferase reporter vector compared with the negative-scramble miRNA control (CmiR0001-MR04) and pMIR-REPORT luciferase vector (Fig. 4). The data indicate the direct binding between WT bta-miR-2899 and the cloned *SPI1* 3′-UTR sequences, through which bta-miR-2899 exerts its inhibitory effect on the upstream luciferase gene. On the other hand, no significant repression effect of MT of bta-miR-2899 was observed on the 3′-UTR of *SPI1* despite the varied dosages of co-transfection in MAC-T, agreeing with the results of TargetScan prediction.

### Association analysis between SNP and milk SCS in dairy cattle

To investigate the genetic effect of a specific SNP (g. 42,198,087 G > A) on mastitis, the association between this SNP and SCS in 322 Chinese Holstein cows was analyzed. The SCS of cows with the AA genotype was significantly (*P* < 0.01) lower than that of cows with the GG and GA genotypes (Table 2), indicating the mutant AA genotype of SNP (g. 42,198,087 G > A) can be a mastitis-resistant genotype. The result further suggests that the SNP is a potential functional marker for the application of selective breeding on mastitis resistance in dairy cattle.

**Table 2:** The genotypic effect of SNP (g. 42,198,087 G > A) on somatic cell score (SCS) in Chinese Holstein cows

Genotype	Genotype count	Allelic frequency(%)	SCS
GG	157	G(87.30)	4.41 ± 0.29^a^
GA	95		3.98 ± 0.27^b^
AA	70	A(12.70)	3.54 ± 0.31^c^

Note: Different superscripts represent significant difference of *P*-value (*P* < 0.05)

## Discussion

During immune and inflammatory responses, the miRNA–argonaute complex interacts with 3′-UTR, 5′-UTR, and coding regions of target genes via complementary binding of miRNA to mRNA. This miRNA-mRNA binding either blocks initiation of translation, induces endonucleolytic cleavage of the target mRNA, or both [19–21]. Polymorphisms and mutations in miRNA precursors and target sites significantly contribute to phenotypic variation including disease susceptibility [22]. Approximately 64% of genome-wide SNPs can modify the binding energy of putative miRNA–mRNA duplexes [23]. Several online tools have been developed to search for miRNA-related SNPs in humans; these tools include miRNASNP v2.0 [24], MicroSNiPer [25], RNAsnp [26], mrSNP [27], and MSDD [28]. However, these tools focus on human miRNA-related SNPs and diseases. In the present study, we used the available bovine SNP, miRNA, and QTL databases to design a customized mRNAsnp software and identified 5252 candidates for miRNA-related and mastitis-related SNPs, which are valuable resources for the studies of miRNA and SNP functions, respectively, and functional markers in the molecular basis of mastitis resistance and molecular breeding in dairy cattle. Of particular note is that favorable associations have been reported between mastitis resistance and several udder conformation traits. For example, higher and more tightly attached udders are associated with lower SCS and less clinical mastitis [29]. Moreover, neutrophil function is very important for mastitis because the neutrophil migration from blood to the site of infection is essential for resolution of most mastitis pathogens. Therefore, genes influencing udder type and neutrophil may also affect mastitis incidence. In the present study, in addition to SCC-, SCS- and CM-related SNPs, we aslo in silico identified the SNPs that are located within the QTLs of udder-type and neutrophil count traits. These candidates are warranted further investigation.

Mastitis occurs when the udder becomes inflamed due to the release of leukocytes into the bovine mammary gland in response to invasion of the teat canal, usually by bacteria [30]. An inflammatory response is then initiated. The invading bacteria multiply and produce toxins, enzymes, and cell wall components which stimulate the production of mediators of inflammation [30]. Milk SCC is a very sensitive biomarker of mammary gland inflammation [31]. Direct selection for mastitis resistance has been considered an inefficient pathway because the mastitis heritability is very low (less than 0.1). In addition, most countries including China do not widely record CM incidences. SCC is routinely monthly recorded in DHI test, and its information is easily available on a large scale. High positive genetic correlations between SCC and clinical mastitis with an average estimate of 0.70. The SCC or SCS reflects both subclinical and clinical mastitis, suggesting as an indicator of mastitis and a tool for selection for mastitis resistance [32]. Identification of SNPs involved in inflammatory response during mastitis could aid to improving herd health through selection of cattle. Therefore, we analyzed the association between SCS and genotypes of SNP to illuminate the relationship between the SNP and mastitis .

Several studies have provided convincing evidence that miRNAs are implicated in the regulation of inflammation and immune [8–14]. Although the miRNA expression and SNPs within the 3′-UTR of inflammation- and immune-related genes as well as their relationships with the risk of bovine mastitis have been extensively investigated, respectively, there are few example showing that mastitis can be affected by the SNPs within miRNAs in dairy cattle.

Based on the predicted result suggested that SPI1 is a direct target of miR-2899. Moreover, increasing evidences have shown that SNPs in miRNA genes including pri-miRNAs, pre -miRNAs and mature miRNAs may influence the processing and target binding of miRNAs. As expected, we confirmed that the SNP (rs109462250, g. 42,198,087 G > A) in the seed region of bta-miR-2899 can influence SPI1 expression by modulating the binding affinities of miRNA and target mRNA and thus cause differences in mastitis resistance. SPI1 is an important member of the ETS transcription factor family, which plays a key role in several steps of the inflammatory pathway [33]. SPI1 is also implicated in transcriptional regulation of several pattern recognition molecules, including Toll-like receptor 4 [34] and TLR9 [35], that trigger inflammation. In macrophages, SPI1 occupies regulatory regions in a large proportion of genes involved in inflammatory pathways, suggesting that SPI1 may be a central regulator of inflammation [17]. Mouse SPI1 gene knockout can also show the development defects of neutrophils, mononuclear cells B cells, and T cells [36]. Therefore, SPI1 gene is considered a candidate factor for immune and inflammation in dairy cattle.

## Conclusions

We identified 5252 miRNA-related SNPs potentially involving mastitis-related traits. We furtherly validated a candidate functional SNP (rs109462250) in the seed region of bta-miR-2899, which is associated with mastitis by affecting the interaction of bta-miR-2899 and SPI1 in Chinese Holsteins. This research is promising, as it may represent an effective functional molecular markers for resistance to mastitis. Our findings will also significantly improve the understanding on miRNA dysfunction in mastitis.

## Methods

### In silico identification of miRNA and QTL-related SNPs

The information on *Bos taurus* miRNAs was downloaded from miRBase version 21 (http://www.mirbase.org/; genome-build-id:UMD3.1). The SNP data were downloaded from National Center for Biotechnology Information (NCBI) dbSNP database (http://www.ncbi.nlm.nih.gov/snp/; Build 133/148, assembly is UMD3.1.1). QTL data were obtained from the database (http://www.animalgenome.org/cgi-bin/QTLdb/BT/; assembly is UMD3.1; it was accessed at 11/01/2016). We designed a perl script, named as miRNAQTLsnp (Additional file 1), to map the SNPs that are simultaneously located within the miRNA sequences and in mastitis-related QTLs. These masitits-relalted QTLs included the Health traits “Mastitis (clinical mastitis, Somatic cell count, Somatic cell score)” and “immune capacity (Neutrophil number, NEUT)” and Udder traits (Udder composite index; Udder depth; Udder height and Teat length) (https://www.animalgenome.org/cgi-bin/QTLdb/BT/ontrait?trait_ID=1464; Genomic locations of QTLs were download by UMD 3.1 assembly). To further narrow down the candidate miRNA-related SNPs, we integrated the differential expression data of miRNAs between the healthy and clinical mastitic cows [10]. We retrived the SNPs that were located within those pre-miRNAs and miRNAs from the Refgene table of UCSC genome browser (http://genome.ucs c.edu/). Then, we used the miRNAQTLsnp to screen the SNPs that were located within the QTLs of 8 traits and pre-miRNAs and miRNAs as the candidate SNPs which were putatively associated with mastitis. We subsequently retrieved the sequences of 3′-UTRs of target genes for the candidate miRNAs. The genomic locations of SNPs were mapped onto pre-miRNAs and 3′-UTRs to obtain the SNPs in miRNA genes. We used the sequence with the reference allele as the wild-type (WT) miRNA and the sequence with mutant allele as the mutant-type (MT) miRNA for each candidate SNP in the bovine miRNA seed regions. Two sequences were considered variant sites, and 25 bp extensions on both sides were retrieved for each SNP in the bovine 3′-UTR. TargetScan (http://www.targetscan.org/vert_50/) [37] and RNA22 [38] with default parameters were used to predict the target sites for the WT and MT sequences. Finally, we obtained the candidate miRNA-related SNPs that are associated with mastitis in dairy cattle. The workflow diagram was shown in Fig. 1.

### Animal samples and bacterial identification

To identify the SNP of bta-miR-2899 and determine the relationship between the SNP and mastitis, 322 blood samples for association analysis were collected from the jugular vein of multiparous Chinese Holstein cows (4–7 years old, 1–4 parity) from 17 sires were selected from five dairy cattle farms participated in the Chinese Dairy Herd Improvement (DHI) program; in dairy farms in Shandong Province. Genomic DNA was extracted according to a previously described publication [39]. DNA samples were stored at − 20 °C for subsequent analysis. Data on milk SCC, an indicator for mastitis status, were provided by the DHI Laboratory of Dairy Cattle Research Center, Shandong Academy of Agricultural Sciences. The SCC values were transformed into SCS values according to the equation: SCS = log _2_^(SCC/100)^ + 3, where SCC is expressed in cells/μL [40].

The relative expression of *SPI1* gene in the mammary gland tissues between the healthy and mastitic cows was investigated. The initial selection of mastitis cow was based on clinical symptoms including redness, swelling, increased heat, and pain. Meanwhile, milk samples from cows with a high monthly SCC were tested for bacteriology and SCC. If one or more quarters had both a positive bacteriology and an SCC ≥500,000 cells/mL, the cow was enrolled and allocated to the mastitic group. If the cow had not clinical sympotoms, and had a negative bacteriology and a milk SCC ≤100,000 cells/mL, it was regarded as the healthy cows. Culturing and identification of pathogens were carried out according to the following procedures within 24 h after sampling. Briefly, a bacteriological loop was used to spread approximately 10 μL of each milk sample or collected fresh mammary gland tissue on blood agar and nutrient broth. The plates were incubated at 37 °C and bacterial growth was examined after 24 h under inverted microscope. Suspected colonies were purified and transferred to nutrient broth and agar slant culture medium. When unusual bacteria were suspected, longer incubation periods or on incubator environment of 10% CO2 were used. If no colony growth was observed within 7 d, samples were considered negative. All isolates were identified based on colony morphologic features, hemolytic characteristics and conventional Gram-staining and visualization under microscope. For the clinical samples, Only *Staphylococcus aureus* was detected in the mammary glands tissues from mastitis-infected cow were used for the present study as described in our previous study [41]. Finally, mammary gland tissues were collected from six healthy and six mastitis-infected Chinese Holstein cows during the first lactation from a commercial slaughter farm. When we prepared these tissue samples, one of the tissue samples was collected and stored in liquid nitrogen for RNA isolation, and another tissue sample was collected for further pathogen identification.

### Detection of SNPs and genotyping

Polymerase chain reaction (PCR) primers (SNP-F: 5′-CATCTGAGCCGGGAATACAG-3′; SNP-R: 5′-GGTCTCTGTTCTGGGGAGAGT-3′; product size = 268 bp; annealing temperature = 64 °C) of bovine miR-2899 (NCBI, AC_000175.1) were designed for PCR amplification using the Primer premier 5 software. The polymorphic PCR-amplified fragments were sent to Beijing Genomics Institute for sequencing. Sequence alignment was performed to screen the SNPs of bta-miR-2899 using DNAMAN version 6.0 [42] and Chromaspro1.41 software (www.technelysium.com.au/chromas.html). The comparison confirmed the SNP (rs109462250, g. 42,198,087 G > A) of bta-miR-2899 in the Holstein cows. The mutant-amplified products generated a natural FauI endonuclease restriction site. Genotyping of the SNP was performed by PCR-restriction fragment length polymorphism assay using 2% agarose gel after FauI restriction enzyme digestion.

### Expression of bovine SPI1 mRNA in mammary gland

Mammary gland tissues from six healthy and six mastitis-infected cows were used for *SPI1* expression analysis. Total RNA extraction and reverse transcription into cDNA were performed according to the previous protocol [43]. The primers (Q-F: 5′- GGAGAGCCATCGGAAGACCT-3′; Q-R: 5′-GGTGCGGATGAAAGTCCCAG-3′; product size = 199 bp) were designed to investigate the relative expression of *SPI1* mRNA in the mammary gland tissues of cows with different genotypes in the SNP (rs109462250, G > A) locus using Q-PCR. The primers of housekeeping internal control β-actin gene were obtained from our previous report [43]. Q-PCR protocol and calculation of relative expression were described as previously reported but with minor modifications [43]. Each 20 μL per well contained 9 μL of 2.5× RealMasterMix/20× SYBR solution (TakaRa), 0.05 μM forward and reverse primers, 9.8 μL ddH_2_O, and 1 μL cDNA or *SPI1*-plasmid DNA. Q-PCR was run on a LightCycler® 480 II (Roche Diagnostics). Each sample was run in triplicate. The Q-PCR conditions were as follows: 50 °C for 2 min and 94 °C for 3 min followed by 40 cycles of 94 °C for 30 s, 55 °C for 40 s, and 68 °C for 15 s. The last stage for the dissociation curve is as follows: 95 °C for 15 s, 60 °C for 15 s, and 95 °C for 15 s.

### Construction of miRNA expression vectors

A specific forward primer (MiR-2899-F: 5′-**GAAGATCT**CATCTGAGCCGGGAATACAG-3′; Bold font represents restriction enzyme site) was designed to include an BgI II restriction site, and a reverse primer (MiR-2899-R: 5′-**CCGGAATTC**GGTCTCTGTTCTGGGGAGAGT-3′) was incorporated with an EcoR I restriction enzyme site using the Primer5 software to amplify bta-miR-2899. The DNA products were extracted and purified using the Gel/PCR Extraction Kit (Biomiga) according to manufacturer’s instructions. The WT (GG) and MT (AA) genotype products were cloned into the pEZX-MR04 vector (OmicsLink), and a plasmid that expresses scramble oligonucleotides was used as a negative control (miR-NC). Plasmids were isolated and purified as our previous protocol [8] and directly sequenced to ensure that the only difference between plasmid sequences was the SNP locus. A specific forward primer (*SPI1*-F: 5′-**CGACGCGT**CCCCGTTGGCCATAGCATTA-3′) including a Hind III restriction site and a reverse primer (*SPI1*-R: 5′-**CCAAGCTT**TCTGGTCAGGCAGTGTCAAC-3′) with a Mlu I restriction site were designed to amplify the 3′-UTR of the *SPI1* gene. Products were cloned into the pMIR- REPORT vector (Promega) to exclude the influence of foreign sequences on 3′-UTR of the *SPI1* gene levels.

Cloning was performed and used to transform Trans5a cells (Invitrogen), which were plated on agar containing 100 mg/mL of ampicillin and incubated at 37 °C overnight. The colonies were screened by PCR for the presence of the insert using two pairs of primers (miR-2899-F, miR-2899-R and *SPI1*-F, *SPI1*-R) as mentioned previously. Positive colonies were cultured in 10 mL lysogeny broth medium (Fisher BioReagents) containing 100 mg/mL of ampicillin and incubated at 37 °C overnight. Plasmids were isolated using the Plasmid Miniprep Kit (Biomiga) according to manufacturer’s instructions, and plasmid DNA was quantified using NanoDrop ND-1000.

### Transient transfection and luciferase reporter assay in cells

MAC-T cells were maintained in Dulbecco’s Modification of Eagle’s Medium (DMEM) supplemented with 10% (v/v) fetal bovine serum, 1% nonessential amino acids, 100 U/mL penicillin, and 100 mg/mL streptomycin. The cells were maintained at 37 °C with 5% CO_2_ and subcultured every other day. All reagents were obtained from GIBCO (https://www.thermofisher.com/) except for those that were specifically assigned. The cells were distributed into a 48-well culture tray containing 3 mL of DMEM complete media per well after subculturing the day before the transfection assay. Although the cells were grown to an 80–85% confluency, the cells were transfected with 100 ng *SPI1* 3′-UTR luciferase expression constructs and 100–300 ng of bta-miR-2899 expression plasmid using LipofectamineTM 2000 (Invitrogen, https://www.thermofisher.com/cn/zh/home/brands/invitrogen.html). These plasmids were cotransfected with 50 ng β-gal for normalization. All experiments were performed with a negative control plasmid (pMIR-REPORT). About 48 h after transfection, the cells were washed once with phosphate-buffered saline for image capturing and lysed in once 65 μL with Reporter Lysis Buffer per well (Promega, http://promega.bioon.com.cn/). Cell lysates from the transfected cells were prepared and assayed for both firefly luciferase and β-gal absorbance values according to the manufacturer’s instructions (Promega). All transfection data are present four repeat independent transfections. The activity was expressed as relative firefly luciferase activity normalized against β-gal absorbance value [8].

### Statistical analyses

All data were analyzed by SPSS software (v.10.0, SPSS Inc., USA). The value of relative miRNA quantity was represented as fold change. Students’ t-tests were used to determine the significance of relative expressions. The effect of bta-miR-2899 genotypes on the activity of *SPI1* was tested by one-way ANOVA. The association analysis between the genotypes of SNP and SCS was analyzed using a general least-square model procedure of the SAS statistical analysis software (SAS Institute Inc., Cary, NC, USA) as described in our previous study [44]. The linear model is expressed as follows: Y_*ijkl*_ = μ + G_*i*_ + P_*j*_ + E_*k*_ + F_*l*_ + e_*ijkl*,_ where Y_*ijkl*_ is the observed value; μ is the overall mean; G_*i*_ is the fixed effect of genotype; P_*j*_ is the fixed effect of age; H_*k*_ is the effect of farm; E_*l*_ is the fixed effect of season; e_*ijkl*_ is the random residual error. Values of SCS were represented with mean ± standard error of the mean (SEM). Values of relative expression of SPI1 mRNA were shown in mean ± standard deviation (SD). A P- value less than 0.05 was regarded as significant.

## Additional files

Additional file 1: miRNAQTLsnp software (WinRAR). The package included 12 files: miRnaQTL.pm, miRNAQTLsnp_get.pl, miRNA-UCSC.txt, pre-miRNA-gff.txt, QTL_CM.txt, QTL_NEUT.txt, QTL_SCC.txt, QTL_SCS.txt, QTL_TLGTH.txt, QTL_UCI.txt, QTL_UDPTH.txt, QTL_UHI.txt. (RAR 121 kb)

Additional file 2: **Table S1**. SNPs within the bovine precursor, mature and seed sequences of microRNAs (.xlsx). (XLSX 429 kb)

Additional file 3: **Table S2**. Candidate miRNA-related SNPs located in the QTLs of 8 traits including CM, SCC, SCS, UDPTH, TLGTH, UCI, UHT and NEUT (8 sheets). (.xlsx). (XLSX 289 kb)

## Abbreviations

3′-UTR: 3′-untranslated regions
CM: Clinical mastitis
MAC-T: Bovine mammary epithelial cells
miRNA: microRNA
NEUT: Neutrophil count
Q-PCR: Quantitative real-time PCR
QTL: Quantitative trait locus
SCS: Somatic cell score
SD: Standard deviation
SEM: Standard error
SNP: Single-nucleotide polymorphism
SPI1: Spi-1 proto-oncogene
TLGTH: Teat length
UCI: Udder composite index
UDPTH: Udder depth
UHT: Udder height
